# Paraneoplastic Inflammatory Arthritis

**DOI:** 10.7759/cureus.2663

**Published:** 2018-05-21

**Authors:** Fawad Rast, Konstantinos Parperis, Surabhi Amar, Mohammed Al-Charakh

**Affiliations:** 1 Internal Medicine, Maricopa Medical Center; 2 Rheumatology, Maricopa Medical Center; 3 Oncology, Maricopa Medical Center

**Keywords:** hiv, lymphoma, diffuse large b cell lymphoma, inflammatory arthritis, inflammatory arthritis, paraneoplastic syndrome, paraneoplastic hypercalcemia, hiv associated lymphoma, hypercalcemia of malignancy

## Abstract

The immunodeficiency virus infection is known to increase the risk of malignancies, including lymphomas. We report a case of a 51-year-old male with a history of human immunodeficiency virus (HIV) infection, well-controlled on antiretroviral treatment, who presented with polyarthritis and hypercalcemia due to an elevated parathyroid-hormone-related peptide. Computer tomography (CT) revealed diffuse lymphadenopathy and a lymph node biopsy revealed large B-cell lymphoma. He was treated and responded well to rituximab, cyclophosphamide, doxorubicin, vincristine, and prednisone (R-CHOP) chemotherapy regimen. Our case highlights the importance of recognizing inflammatory arthritis as an initial manifestation of occult malignancy like large B-cell lymphoma, as the arthritis preceded his eventual diagnosis of lymphoma by several months.

## Introduction

Human immunodeficiency virus (HIV) is a cytopathic retrovirus and the cause of acquired immunodeficiency syndrome (AIDS), a chronic viral infection that has been associated with a higher risk of cancer, including non-Hodgkin lymphoma. Large B-cell lymphoma is the most common lymphoma, accounting for 25% of the non-Hodgkin’s lymphomas, with an incidence reported to be seven cases for 100,000 persons per year in the United States [1]. We report a case of a patient with HIV infection on antiretroviral treatment (ART) who presented with symptoms of inflammatory arthritis that did not respond to immunosuppression. Subsequently, the patient developed hypercalcemia due to an elevated parathyroid-hormone-related peptide and lymphadenopathy. Further work-up with a lymph node biopsy implicated large B-cell lymphoma as the etiology of the paraneoplastic syndrome of arthritis and elevated calcium.

## Case presentation

A 51-year-old male with a history of well-controlled HIV infection on anti-retroviral treatment presented to the rheumatology clinic for the evaluation of a two-month history of symmetric polyarthritis involving bilateral knees, ankles, and feet. The joint was aching, and the pain was present at rest and with activity. The pain is associated with joint swelling and morning stiffness lasting approximately one hour. He was previously treated with a two-week course of prednisone 20 mg daily without any improvement of his symptoms. The patient was diagnosed with HIV at the age of 33 and he was on ART regimen, including efavirenz 600 mg, emtricitabine 200 mg, and tenofovir 300 mg. His most recent CD4 count was 382 with an undetectable HIV viral load.

The patient’s vital signs were within normal limits. The physical examination was remarkable for tenderness to palpation in his feet and knees. There was ankle synovitis with moderate effusion, limiting the range of motion. Cervical lymph nodes were enlarged, mobile, and non-tender. There was no other lymphadenopathy or hepatosplenomegaly on examination.

**Figure 1:**
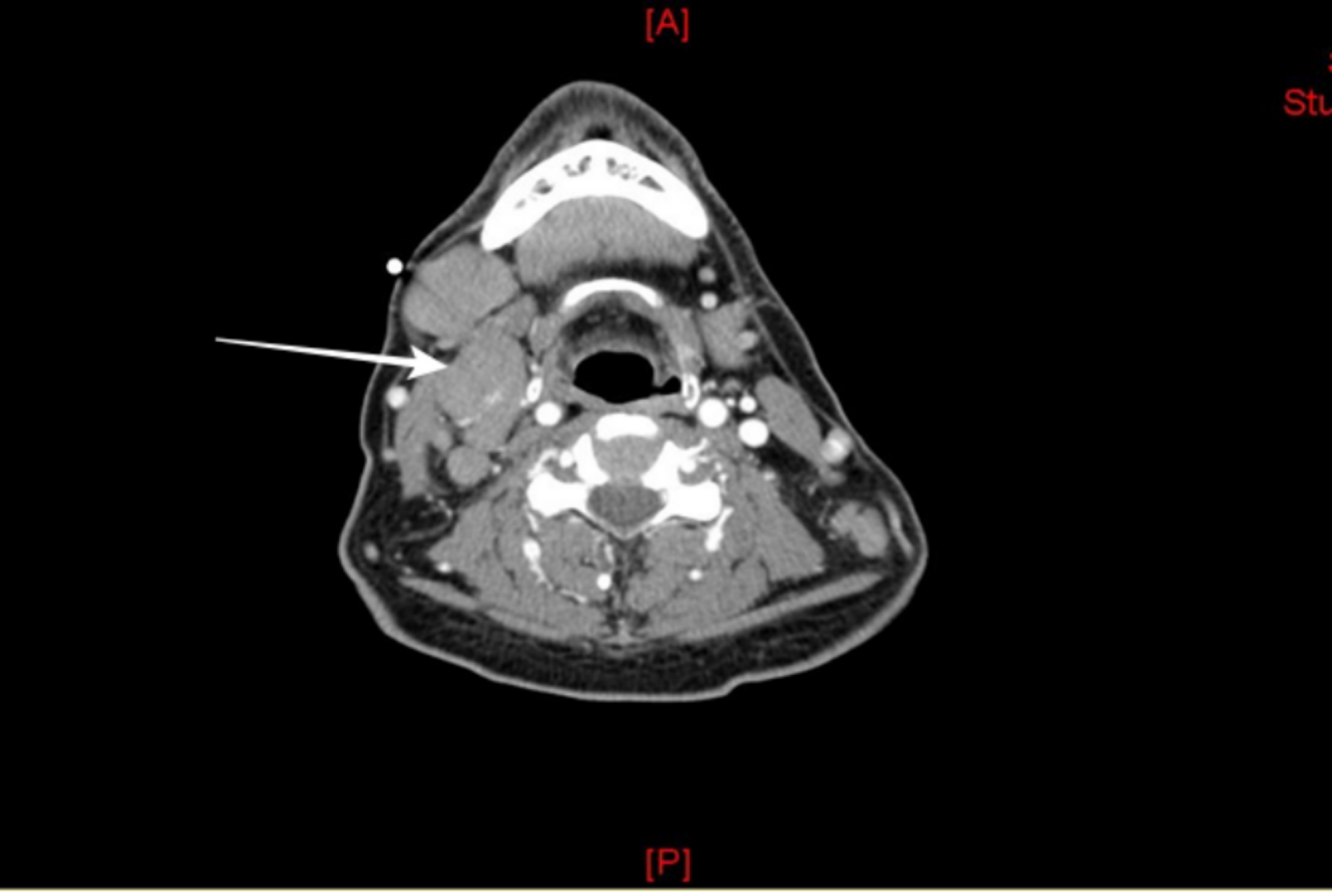
Computed tomography of cervical soft tissue Diffusely enlarged lymph nodes; the largest, most superficial, and most amenable to percutaneous biopsy measuring 2.3 cm in the short axis diameter in the right submandibular region.

Radiographs of both knees revealed bilateral large suprapatellar effusions. Left knee arthrocentesis was performed and demonstrated a white blood count of 27,900 cells/mm3 (0-200 cells/mm3) with no crystals. Erythrocyte sedimentation rate and C-reactive protein were elevated at 54 mm/hr and 122 mg/L, respectively. Other studies, including synovial fluid gram stain, cultures, antinuclear antibody, rheumatoid factor (RF), cyclic citrullinated peptide antibody, and rapid plasma reagin were all negative. The patient’s symptoms did not improve with a trial of a higher dose of prednisone - 40 mg daily and intramuscular triamcinolone injection. The addition of sulfasalazine and methotrexate did not provide any relief to the patient’s symptoms. He developed progressive swelling of his cervical lymph nodes, decreased appetite, nausea, and recurrent emesis. The patient lost approximately 12 kg (26 lb) over the period of three months. On initial evaluation, the calcium level was normal but eight weeks later, his calcium level increased to 13 mg/dl. Computed tomography revealed extensive lymphadenopathy involving the cervical lymph nodes (Figure 1).

**Figure 2:**
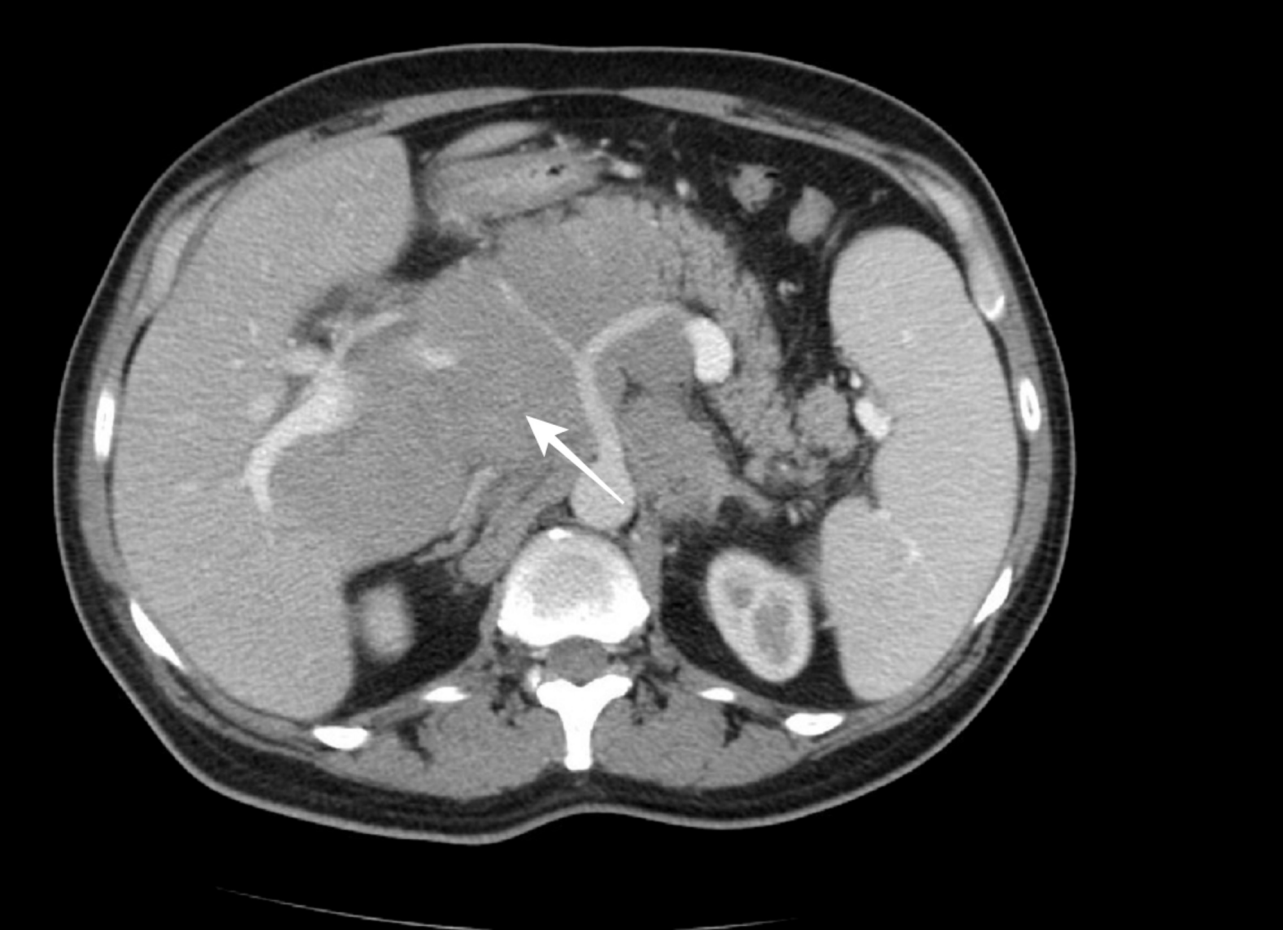
Computed tomography axial abdominal Computed tomography demonstrates extensive lymphadenopathy, resulting in a mass effect on the medial aspect of the liver.

**Figure 3:**
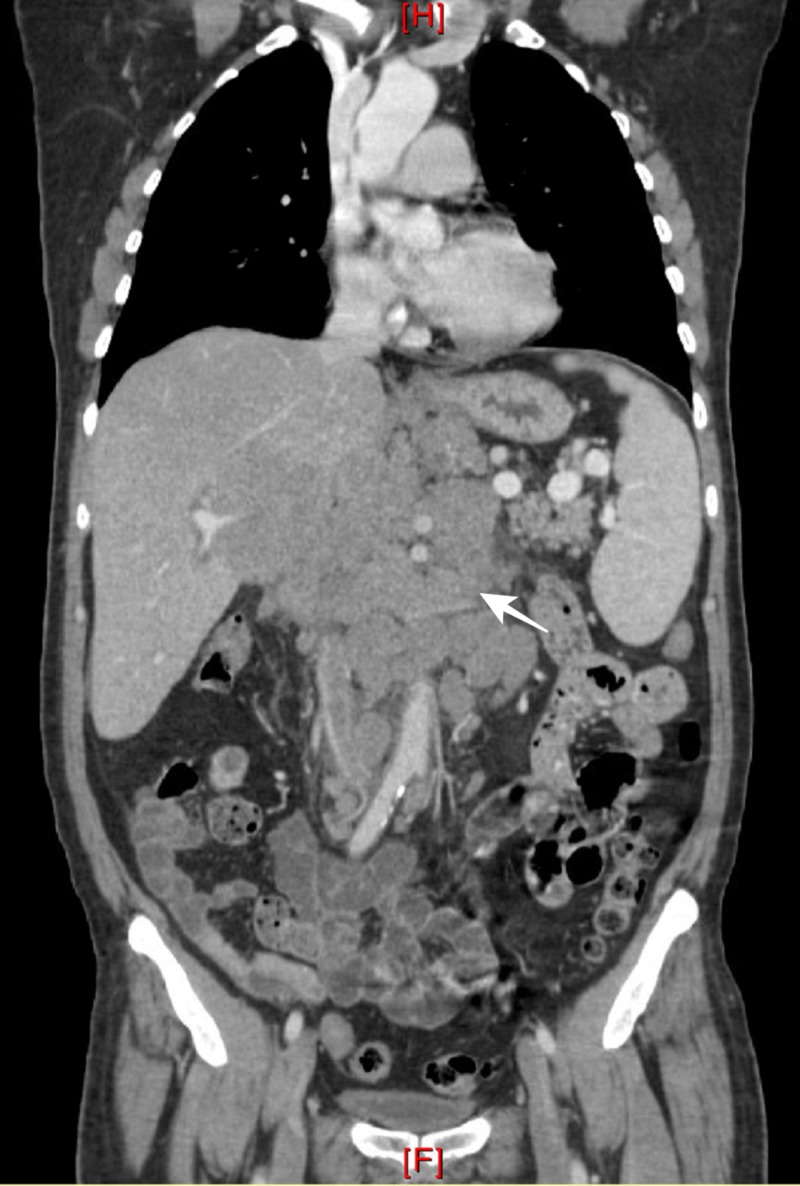
Computed tomography coronal view Extensive lymphadenopathy throughout the chest, abdomen, and pelvis with a large conglomerate of lymph nodes at the mesenteric root, encasing the celiac artery and portal venous confluence with near complete effacement of the portal vein.

Imaging studies also demonstrated further lymphadenopathy in the mediastinum, abdomen, and pelvis with the largest measuring 9.5 x 15 cm, as well as splenomegaly (Figures 2-3). There was no evidence of any bony lytic or sclerotic lesions. The patient gradually developed an altered mental status, requiring hospital admission. Initial admission laboratory studies were significant for a calcium level of 19.3 mg/dL, creatinine of 2.04 mg/dL, and uric acid level of 17.6 mg/dL.

The patient’s overall clinical presentation was consistent with tumor lysis syndrome from hematologic malignancy. He was treated in the intensive care unit for severe hypercalcemia and he received intravascular fluids, zoledronic acid, and calcitonin. This resulted in an improvement in renal function and hypercalcemia.

**Figure 4:**
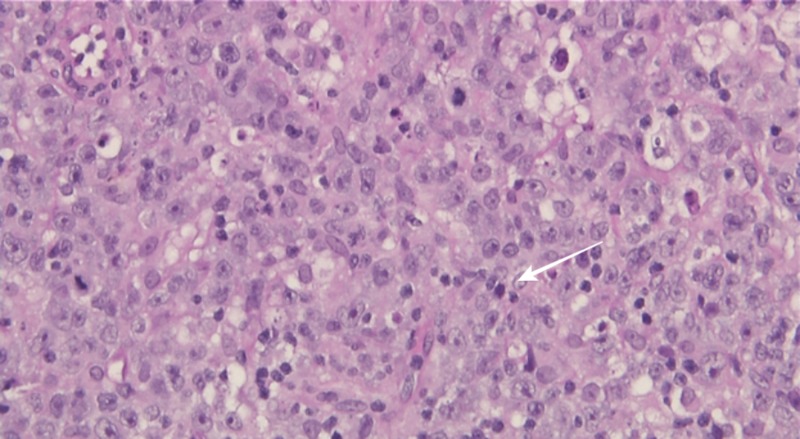
Right submandibular lymph node core needle biopsy Showing the effacement of the lymphoid architecture by a diffuse sheet-like proliferation of large lymphoid cells with enlarged irregular nuclei.

Workup for the hypercalcemia showed an elevated parathyroid-hormone-related peptide level of 6.1 pmol/ml (normal level < 2.5), low parathyroid hormone at the level of 6.4 pg/ml (normal 10-65), and normal levels of 1,25-hydroxyvitamin D and 25-hydroxyvitamin D, supporting a paraneoplastic etiology for the hypercalcemia. A right submandibular lymph node biopsy was performed that yielded the effacement of the lymphoid architecture by a diffuse sheet-like proliferation of large lymphoid cells that had enlarged irregular nuclei with vesicular chromatin and prominent nucleoli (Figure 4).

Immunoperoxidase stains showed that the neoplastic cells were strongly and diffusely positive for CD20, CD79A, BCL2, and LCA and negative for CD10, BCL-6, CD5, and cyclin D1. These findings were consistent with diffuse large B-cell lymphoma (DLBCL). Bone marrow biopsy and cerebrospinal fluid cytology excluded bone marrow and central nervous system involvement. Based on these results, our patient was diagnosed with HIV-associated diffuse large B cell lymphoma (DLBCL).

## Discussion

Malignancies can present with a wide variety of paraneoplastic features, including rheumatic manifestations and hypercalcemia. In patients with DLBCL, hypercalcemia can occur due to the secretion of the parathyroid-hormone-related protein and it is considered an unusual feature of the disease [2]. Rheumatic manifestations of malignancies include paraneoplastic polyarthritis, tumor invasion of the joint or bone, hypertrophic osteoarthropathy, and vasculitis [3].

Paraneoplastic polyarthritis has been reported in association with both solid tumors (lung, breast, colon, stomach, and kidney) as well as hematological malignancies (leukemias and lymphomas) [4]. Although the articular symptoms are rare, they might be the initial presentation of the disease and may precede the diagnosis of the underlying malignancy by months [4].

Arthritis may present as an asymmetric polyarthritis involving predominantly the lower extremities or symmetric polyarthritis affecting small and large joints and may resemble rheumatoid arthritis [5]. It is characterized by the absence of high titer of rheumatoid factor, (occasionally low titer has been reported), rheumatoid nodules, a family history of rheumatoid arthritis, and radiographic findings like erosive joint disease [3-7]. In addition, most patients are male, older than 50, have an explosive onset of the disease, and a close relationship between the onset of arthritis and the diagnosis of the malignancy [6]. Data regarding synovial fluid findings in paraneoplastic arthritis are limited. Previous studies reported a synovial fluid cell count ranging between 3,500 and 55,000/mm3 in patients diagnosed with lymphoma [8-9].

The pathogenesis of paraneoplastic arthritis remains elusive. Circulating cytokines and immune complexes, abnormalities in cell-mediated immunity, and antigenic cross-reactivity between the tumor and synovium have previously been proposed as underlying mechanisms for this condition [10].

The use of steroids or conventional immunosuppression to treat arthritis can lead to minimal or partial treatment of lymphomas, leading to temporary resolution of the symptoms and a further delay in the diagnosis of the underlying malignancy. The treatment of the underlying cancer usually results in a resolution of arthritic symptoms, which can recur at the time of cancer relapse. Our patient was treated with six cycles of R-CHOP (rituximab, cyclophosphamide, doxorubicin, vincristine, and prednisone) chemotherapy. After the first cycle of chemotherapy, the patient had complete resolution of his rheumatologic and constitutional symptoms. Post-chemotherapy scans revealed a good partial response with complete resolution of thoracic and cervical nodes and splenomegaly but residual abdominal nodes (6.4 x 3.8 cm). At the time of writing this manuscript, the patient was receiving radiation treatments to the residual nodes.

## Conclusions

In our case, lymphoma was masquerading as polyarthritis; therefore, it is critical for health-care providers to consider inflammatory polyarthritis not responding to immunosuppression as an early manifestation of occult lymphoma, which may lead to earlier diagnosis and treatment.
